# Exposure to secondhand smoke among school-going adolescents in Malaysia: Findings from the tobacco and e-cigarettes survey among Malaysian adolescents (TECMA)

**DOI:** 10.18332/tid/128622

**Published:** 2020-11-20

**Authors:** Miaw Y. J. Ling, Kuang H. Lim, Wan Shakira R. Hasani, Halizah M. Rifin, Nur Liana A. Majid, Tania G. R. Lourdes, Thamil A. Saminathan, Ying Y. Chan, Ahzairin Ahmad, Hasimah Ismail, Muhammad Fadhli M. Yusoff

**Affiliations:** 1 Institute for Public Health, National Institutes of Health, Shah Alam, Malaysia; 2 Institute for Medical Research, Kuala Lumpur, Malaysia

**Keywords:** secondhand smoke, home, school-going adolescents, outside the home, nationwide study

## Abstract

**INTRODUCTION:**

Many studies have revealed that exposure to secondhand smoke (SHS) substantially increases the risk of smoking related diseases especially among the vulnerable groups, yet data on the location of SHS exposure among youth in Malaysia are still lacking. The study aims to describe the prevalence and factors associated with SHS exposure at home, outside the home, and inside the school among school-going adolescents in Malaysia.

**METHODS:**

We derived the data from the TECMA study, which used a cross-sectional study design and multi-stage sampling method to obtain a representative sample of school-going adolescents aged 11–19 years in Malaysia in 2016. Data were collected through a self-administered approach using a pre-validated standard questionnaire. Descriptive and multivariate analyses were used to analyze the data, and results are presented as adjusted odds ratio (AOR) with 95% confidence interval (95% CI).

**RESULTS:**

SHS exposure for the past seven days was higher outside the home (51.2%; 95% CI: 49.2–53.2) compared to at home (37.8%; 95% CI: 35.8–39.9) while 27.3% (95% CI: 25.1–29.5) of school-going adolescents reported exposure to SHS inside the school in the past one month. In the regression analyses, older adolescents, those of Malay and Bumiputra Sarawak ethnicities, adolescents from rural areas and current smokers had higher likelihood of exposure to SHS at home, outside home and inside the school. Our study also found that adolescents who were current smokers had higher odds of being exposed to SHS at home (AOR=2.87; 95% CI: 2.57–3.21), outside the home (AOR=3.46; 95% CI: 3.05–3.92) and in the school (AOR=2.25; 95% CI: 2.01–2.51).

**CONCLUSIONS:**

Health promotion measures should target parents/guardians and household members to reduce SHS exposure among adolescents. In addition, smoke-free regulation should be fully enforced in school. Furthermore, more public places should be designated non-smoking areas to reduce SHS exposure and denormalize smoking behavior.

## INTRODUCTION

Many scientific studies have demonstrated that secondhand smoke (SHS) exposure is associated with increased morbidity and mortality^1^. Globally, 10.9 million disability-adjusted life years (DALYs) and about 0.6 million premature deaths were caused by diseases related to SHS exposure, with 28% of these deaths in children (165000 and 1100 deaths attributed to lower respiratory infections and asthma, respectively)^2^. Children are vulnerable to the negative health effects associated with SHS exposure due to their less developed immune systems, rapid breathing and due to their small size, which cause them to absorb relatively more pollutants^3^. Furthermore, adolescents who were exposed to SHS had higher risk of susceptibility to smoking and smoking initiation^4^.

The Ministry of Health has developed the National Strategic Plan on Tobacco Control (NSPTC) 2015–2020 and one of the goals was to protect Malaysians from secondhand smoke exposure^5^. In order to achieve the goals, many efforts have been undertaken such as the expansion of gazetted smoke-free areas through the amendment of the Control of Tobacco Product Regulation 2004 (CTPR 2004)^6^, which is in line with the provisions of Article 8 of the WHO Framework Convention on Tobacco Control. Under provisions of the CTPR 2004, a total of 23 types of places have been gazetted as smoke-free areas in Malaysia, including all eateries, which came into force recently. With the smoking ban at eateries in place, offenders will face a fine of up to MYR 10000 (Malaysian Ringgit about 2400 US$) or two years’ jail, while restaurant owners will face a fine of up to MYR 3000 or 6 months’ jail if they allow smoking on their premises^7^. The Ministry of Health is also committed to expand the smoking ban gradually in pubs and casinos, which have not been gazetted as smoke-free areas^7^. Also, the Malaysian Health Promotion Board (MySihat) together with other partner organizations have been promoting and supporting the smoke-free city initiatives in the state of Malacca (Melaka Bebas Asap Rokok; MBAR), followed by Johor (Johor Bebas Asap Rokok; JBAR), Pulau Pinang (Pulau Pinang Bebas Asap Rokok; PENBAR), Kelantan (Inisiatif Kelantan Bebas Asap Rokok; IKBAR) and Terengganu (Terengganu Bebas Asap Rokok; TBAR) with a total of 33 areas being gazetted as non-smoking zones^8^.

In addition, school premises where students spent a significant amount of their time have been gazetted as smoke-free areas under the CTPR 2004, and the Ministry of Education has issued a circular to all public school administrators to ensure adherence to the stipulated regulation^9^. Also, the Ministry of Health has introduced school-based health promotion programs for school children such as the young doctors’ program and ‘program sihat untuk remaja’ (PROSTAR) to facilitate health promotion such as education against tobacco^10^. Furthermore, smoke-free homes have been promoted through a community interventional program (KOSPEN), which was implemented by the Ministry of Health and Ministry of Rural Development in selected localities to reduce SHS exposure at home^11^. Enhancing health promotion programs, to increase awareness of the health hazards of SHS exposure, that target both adults and adolescents is one of the strategies to reduce SHS exposure.

Studies have highlighted that 40% of children reported SHS exposure globally and the risk of exposure was higher among girls, older adolescents, smokers, those whose parents smoked and those whose peers smoked^2,12^. Previous studies on SHS exposure among adolescents in Malaysia have reported prevalence ranging from 41.5% to 56.4% and several factors such as being older, male, Malay, smoker, and having parent/s who smoke were found to be associated with SHS exposure^13,14^. However, these studies did not specify the areas/localities of exposure, which thwarts the formulation of appropriate policies to address the problem effectively as different approaches are required for different localities. In addition, some of the studies were conducted locally that involved a non-representative sample of Malaysian school-going adolescents.

The latest studies on SHS exposure among Malaysian school-going adolescents were conducted in 2013^14^. As time passed, the smoking landscape in Malaysia might have changed due to the extension of smoke-free areas that may have resulted in the displacement of smoking into homes, encouragement of anti-smoking norms, changes in the types of tobacco products used and increasing awareness of SHS hazards among the Malaysian population. Furthermore, the introduction and implementation of anti-smoking programs and policies might have changed the prevalence and associated factors of SHS exposure in Malaysia. Thus, our study aims to determine the prevalence and associated factors of SHS exposure in various localities (at home, outside the home and inside the school) among school-going adolescents in Malaysia using the latest data from a national survey conducted among Malaysian school-going adolescents in 2016.

## METHODS

### Study setting, design and participants

The data used in this study were obtained from the tobacco and e-cigarette survey among Malaysian adolescents (TECMA) 2016^15^, a national school-based study that investigate cigarette smoking, e-cigarette and shisha use among school-going adolescents in Malaysia. The TECMA was a cross-sectional study that used a two-stage stratified cluster sampling design to select a representative sample of school-going adolescents aged 11–19 years in Malaysia. Data were collected from respondents using a self-administered approach (filling in answers on an OMR form). Prior to the study, respondents were given a detailed explanation about the survey and were informed that their participation was voluntary. Respondents were also assured that their information was confidential and would only be used for study purposes. Only respondents who gave their consent and also obtained the consent of their parents/guardians were allowed to participate in the study. The study protocol was approved the Ministry of Education Malaysia, whereas approval to recruit the students as participants was given by the individual schools and their respective districts, and the State Education Department. Ethical approval for the TECMA was obtained from the Medical Research & Ethics Committee, Ministry of Health Malaysia (NMRR-16-108-28789).

### Sample size determination

Sampling frame used in the TECMA was provided by the Ministry of Education. Malaysia was stratified into 15 states, followed by the division of schools into urban and rural areas for each state, based on the classification of Ministry of Education. The school, as the primary sampling unit, was randomly selected from each state using systematic probability sampling proportional to the enrolment of students in each school. The second stage of sampling involved the selection of class at the selected school using the simple random sampling method. All students from the selected classes were invited to participated in the study. Sample size was determined using a prevalence of e-cigarette usage of 3%^16^, design effect of 1.5 and expected non-response rate of 20%. A total of 13980 students from 138 schools from 15 states were required for the survey. Detail description of the study can be found elsewhere^15^.

### Instrument

The questionnaire used in this study was adapted from the Global Youth Tobacco Survey (GYTS) questionnaire. In order to suit the local situation, additional items were also included by the subject matter experts from the research institutes and Ministry of Health Malaysia.

### Validity and reliability

Items adapted from the GYTS underwent forward and backward translation by content and language expert teams. The instrument was pretested among school-going adolescents in selected schools to establish face validity.

### Measurement

The dependent variables in our study (exposure to SHS at home, outside the home and inside the school) were measured using the following items: a) ‘During the past seven days, on how many days has anyone smoked inside your home, in your presence?’ (responses: 0, 1–2, 3–4, 5–6, or 7 days); b) ‘During the past seven days, on how many days were you exposed to cigarette smoke from other people in places other than your home?’ (responses: 0, 1–2, 3–4, 5–6, or 7 days); and c) ‘During the past 30 days, have you ever seen anyone smoking inside the school compound?’ (responses: yes, no). Those who answered non-zero to the first two items were classified as being exposed to SHS at home and outside the home, respectively, while those who answered ‘yes’ to the third item were classified as being exposed to SHS inside the school^12,17^.

The independent variables in this study were gender (male, female), age (≤12, 13–15, ≥16 years), ethnicity (Malay, Chinese, Indian, Bumiputra Sabah, Bumiputra Sarawak, other), school location (urban, rural), current smoking status (yes, no), and perceive SHS as harmful (yes, no).

The categorization of school location (urban, rural) was based on the classification of the Ministry of Education. Current smoker was defined as someone who smoked any tobacco product during the past 30 days. Perception of SHS as harmful was measured using the following item: ‘Do you think the smoke from other people’s cigarette smoking is harmful to you?’ (responses: yes, no). Those who answered ‘yes’ were considered to perceive SHS as harmful.

### Statistical analyses

Data entry included the scanning of an OMR form into excel format, where the data were exported to SPSS statistical software for analysis. Data were cleaned and sample weight was calculated (taking into account the complex sample design and response rate) to ensure that estimates can be produced for the target population. Descriptive statistics were used to describe the characteristics of respondents. The prevalence of SHS exposure at home, outside the home, and inside the school were estimated. Simple logistic regression analysis was used to test the associations between all categorical independent variables with exposure to SHS. Variables with p<0.25 were included into the multiple logistic regression analysis to determine the influence of each variable on SHS exposure at home, outside the home and inside the school (except for gender and perception that SHS is harmful, as they were important variables with significant associations reported in other studies). A two-way interaction analysis between the independent variables in the models were carried out and no significant two-way interaction was detected (p>0.05) in all models. Diagnostic testing to assess the goodness-of-fit was conducted to ensure the fit of the logistic regression model for individual cases or covariates. SPSS version 21 software was used to carry out all statistical analysis, at 95% confident interval.

## RESULTS

Table 1 shows the sociodemographic characteristic of the respondents. The sample consisted of almost equal proportions of males (50.1%) and females (49.9%). About two-thirds of the respondents were Malay (70.4%), followed by Chinese (13.4%). More than half of the respondents were schooling in urban areas. More than one-third of the respondents were aged 13–15 years (40.2%), followed by ≤12 years (31.5%) and ≥16 years (28.3%).

**Table 1:** Sociodemographic characteristics and current smoking status among school-going adolescents in Malaysia, 2016 (N=13136)

*Characteristics*	*Sample (n)*	*Percentage (%)*
**Gender**		
Male	6582	50.1
Female	6554	49.9
**Age** (years)		
≤12	4138	31.5
13–15	5278	40.2
≥16	3720	28.3
**Ethnicity**		
Malay	9243	70.4
Chinese	1764	13.4
Indian	748	5.7
Bumiputra Sabah	545	4.2
Bumiputra Sarawak	447	3.4
Other	385	2.9
**School location**		
Urban	7688	58.5
Rural	5448	41.5
**Current smoker**		
Yes	1807	13.8
No	11329	86.2

Table 2 presents the prevalence of SHS exposure at home, outside the home and inside the school. SHS exposure for the past seven days was higher outside the home (51.2%; 95% CI: 49.2–53.2) compared to at home (37.8%; 95% CI: 35.8–39.9) while about a quarter (27.3%; 95% CI: 25.1–29.5) of school-going adolescents reported exposure to SHS inside the school in the past one month. The prevalence of SHS exposure increased significantly with increasing age at all places. In addition, SHS exposure was significantly higher among Malay and Bumiputra Sarawak as well as among those who were current smokers at all places (at home, outside the home and inside the school). Also, adolescents schooling in rural areas had significantly higher SHS exposure at home and outside the home, while male adolescents had significantly higher SHS exposure in the school.

**Table 2:** Prevalence of secondhand smoke exposure among school-going adolescents in Malaysia by sociodemographic characteristics, 2016 (N=13136)

*Characteristics*	*Exposure to SHS at home*			*Exposure to SHS outside the home*			*Exposure to SHS in the school*		
	*n*	*%*	*95% CI*	*n*	*%*	*95% CI*	*n*	*%*	*95 % CI*
**Overall**	4644	37.8	35.8–39.9	6680	51.2	49.2–53.2	3714	27.3	25.1–29.5
**Gender**									
Male	2352	37.6	35.2–40.1	3527	53.2	50.9–55.5	2251	31.9	29.2–34.7
Female	2292	38.1	35.3–40.9	3153	49.1	46.7–51.5	1463	22.4	20.1–24.9
**Age** (years)									
≤12	1360	32.9	29.7–36.3	1774	43.2	40.5–46.0	854	20.9	18.1–24.1
13–15	1890	40.2	36.9–43.7	2722	53.8	50.2–57.5	1510	27.6	23.7–31.9
≥16	1394	41.5	38.0–45.0	2184	59.3	56.2–62.4	1350	36.5	32.5–40.7
**Ethnicity**									
Malay	3582	41.6	39.0–44.2	5140	56.1	53.6–58.5	2773	28.2	25.4–31.2
Chinese	361	21.3	18.6–24.2	570	33.1	28.5–38.0	369	20.9	17.3–25.1
Indian	119	16.8	13.5–20.7	241	32.1	26.4–38.3	178	25.4	20.9–30.5
Bumiputra Sabah	229	44.2	38.6–50.5	300	55.5	47.5–63.3	144	23.7	19.3–28.8
Bumiputra Sarawak	218	48.5	42.5–54.5	263	59.3	53.8–64.6	157	39.3	27.5–52.4
Other	133	36.2	28.4–44.8	164	40.6	32.6–49.0	91	23.5	18.3–29.7
**School location**									
Urban	2392	31.1	28.6–33.6	3730	44.9	42.6–47.2	2063	25.1	22.7–27.7
Rural	2252	43.4	40.7–46.2	2950	56.5	53.6–59.4	1651	29.1	25.7–32.7
**Current smoker**									
Yes	1050	62.1	57.8–66.2	1398	76.5	73.5–79.2	897	46.9	42.6–51.3
No	3594	33.8	31.8–35.8	5282	47.0	45.0–49.0	2817	24.0	21.9–26.2
**Perceive secondhand smoke as harmful**									
Yes	3211	37.9	35.6–40.2	4792	53.5	51.2–55.8	2608	27.5	25.2–29.9
No	1430	37.6	34.5–40.9	1884	46.5	43.3–49.8	1104	26.8	24.0–29.8

Table 3 presents the simple logistic regression analysis for factors associated with SHS exposure among Malaysian adolescents. Multiple logistic regression analysis (Table 4) revealed that male adolescents were at higher risk of SHS exposure outside the home (AOR=1.08; 95% CI: 1.01–1.17) and at school (AOR=1.62; 95% CI: 1.49–1.77) but less likely to be exposed to SHS in the home (AOR=0.85; 95% CI: 0.79–0.92). The odds of being exposed to SHS were also higher among older adolescents at home (AOR=1.17; 95% CI: 1.06–1.29), outside the home (AOR=1.91; 95% CI: 1.73–2.10) and in the school (AOR=2.26; 95% CI: 2.03–2.51) compared with younger adolescents. Adolescents of Malay and Bumiputra Sarawak ethnicity were more likely to be exposed to SHS at home (Malay: AOR=2.10; 95% CI: 1.85–2.38; and Bumiputra Sarawak: AOR=2.96; 95% CI: 2.36–3.71), outside the home (Malay: AOR=2.50; 95% CI: 2.24–2.80; and Bumiputra Sarawak: AOR=2.85; 95% CI: 2.28–3.56) and in the school (Malay: AOR=1.58; 95% CI: 1.39–1.80; and Bumiputra Sarawak: AOR=1.98; 95% CI: 1.56–2.51) compared with Chinese adolescents. Adolescents who were from rural areas were more likely to be exposed to SHS at home (AOR=1.35; 95% CI: 1.26–1.46), outside the home (AOR=1.08; 95% CI: 1.01–1.17) and in the school (AOR=1.11; 95% CI: 1.02–1.20). Our study also found that adolescents who were current smokers had higher odds of being exposed to SHS at home (AOR=2.87; 95% CI: 2.57–3.21), outside the home (AOR=3.46; 95% CI: 3.05–3.92) and in the school (AOR=2.25; 95% CI: 2.01–2.51). In addition, adolescents who did not perceive SHS as harmful were less likely to be exposed to SHS outside the home (AOR=0.77; 95% CI: 0.71–0.84) and in the school (AOR=0.91; 95% CI: 0.83–0.99).

**Table 3:** Simple logistic regression analysis for factors associated with secondhand smoke exposure among school-going adolescents in Malaysia, 2016 (N=13136)

*Variable*	*Exposure to SHS at home*		*Exposure to SHS outside the home*		*Exposure to SHS in the school*	
	*OR (95% CI)*	*p*	*OR (95% CI)*	*p*	*OR (95% CI)*	*p*
**Gender**						
Male	1.03 (0.96–1.11)	0.354	1.25 (1.16–1.33)	<0.001	1.81 (1.68–1.96)	<0.001
Female (Ref.)	1.00		1.00		1.00	
**Age** (years)						
≤12 (Ref.)	1.00		1.00		1.00	
13–15	1.14 (1.05–1.24)	0.003	1.42 (1.31–1.54)	<0.001	1.54 (1.40–1.70)	<0.001
≥16	1.22 (1.12–1.34)	<0.001	1.89 (1.73–2.07)	<0.001	2.19 (1.98–2.42)	<0.001
**Ethnicity**						
Malay	2.46 (2.18–2.78)	<0.001	2.63 (2.36–2.93)	<0.001	1.62 (1.44–1.84)	<0.001
Chinese (Ref.)	1.00		1.00		1.00	
Indian	0.74 (0.59–0.92)	0.008	1.00 (0.83–1.20)	0.963	1.18 (0.96–1.45)	0.110
Bumiputra Sabah	2.82 (2.29–3.46)	<0.001	2.57 (2.11–3.12)	<0.001	1.36 (1.09–1.70)	0.007
Bumiputra Sarawak	3.70 (2.97–4.60)	<0.001	2.99 (2.42–3.71)	<0.001	2.05 (1.63–2.57)	<0.001
Other	2.05 (1.61–2.61)	<0.001	1.55 (1.24–1.95)	<0.001	1.17 (0.90–1.53)	0.229
**School location**						
Urban	1.00		1.00		1.00	
Rural	1.56 (1.45–1.68)	<0.001	1.25 (1.17–1.34)	<0.001	1.19 (1.10–1.28)	<0.001
**Current smoker**						
Yes	2.99 (2.70–3.31)	<0.001	3.91 (3.48–4.39)	<0.001	3.00 (2.71–3.32)	<0.001
No	1.00		1.00		1.00	
**Perceive secondhand smoke as harmful**						
Yes (Ref.)	1.00		1.00		1.00	
No	1.04 (0.96–1.12)	0.351	0.82 (0.76–0.88)	<0.001	0.96 (0.89–1.05)	0.386

OR: odds ratio.

**Table 4:** Multiple logistic regression analysis for factors associated with secondhand smoke exposure among school-going adolescents in Malaysia, 2016 (N=13136)

*Variable*	*Exposure to SHS at home ^a^*		*Exposure to SHS outside the home ^b^*		*Exposure to SHS in the school ^c^*	
	*AOR (95% CI)*	*p*	*AOR (95% CI)*	*p*	*AOR (95% CI)*	*p*
**Gender**						
Male	0.85 (0.79–0.92)	<0.001	1.08 (1.01–1.17)	0.043	1.62 (1.49–1.77)	<0.001
Female (Ref.)	1.00		1.00		1.00	
**Age** (years)						
≤12 (Ref.)	1.00		1.00		1.00	
13–15	1.09 (1.00–1.19)	0.053	1.39 (1.27–1.51)	<0.001	1.52 (1.37–1.67)	<0.001
≥16	1.17 (1.06–1.29)	0.002	1.91 (1.73–2.10)	<0.001	2.26 (2.03–2.51)	<0.001
**Ethnicity**						
Malay	2.10 (1.85–2.38)	<0.001	2.50 (2.24–2.80)	<0.001	1.58 (1.39–1.80)	<0.001
Chinese (Ref.)	1.00		1.00		1.00	
Indian	0.69 (0.55–0.87)	0.002	0.98 (0.81–1.18)	0.847	1.16 (0.94–1.43)	0.160
Bumiputra Sabah	2.10 (1.70–2.60)	<0.001	2.07 (1.69–2.54)	<0.001	1.11 (0.88–1.39)	0.403
Bumiputra Sarawak	2.96 (2.36–3.71)	<0.001	2.85 (2.28–3.56)	<0.001	1.98 (1.56–2.51)	<0.001
Other	1.80 (1.41–2.30)	<0.001	1.57 (1.24–1.99)	<0.001	1.22 (0.93–1.61)	0.146
**School location**						
Urban (Ref.)	1.00		1.00		1.00	
Rural	1.35 (1.26–1.46)	<0.001	1.08 (1.01–1.17)	0.036	1.11 (1.02–1.20)	0.014
**Current smoker**						
Yes	2.87 (2.57–3.21)	<0.001	3.46 (3.05–3.92)	<0.001	2.25 (2.01–2.51)	<0.001
No (Ref.)	1.00		1.00		1.00	
**Perceive secondhand smoke as harmful**						
Yes (Ref.)	1.00		1.00		1.00	
No	0.97 (0.89–1.05)	0.462	0.77 (0.71–0.84)	<0.001	0.91 (0.83–0.99)	0.030

AOR: adjusted odds ratio; adjusted for gender, age group, ethnicity, school location, current smoking status and perception of SHS as harmful.

a Multicollinearity and interactions were checked and not found. Hosmer–Lemeshow test p=0.063; Classification table (overall correctly classified percentage = 67%); ROC curve = 65%.

b Multicollinearity and interactions were checked and not found. Hosmer–Lemeshow test p=0.089; Classification table (overall correctly classified percentage = 62%); ROC curve = 67%.

c Multicollinearity and interactions were checked and not found. Hosmer–Lemeshow test p=0.092; Classification table (overall correctly classified percentage = 73%); ROC curve = 65%.

## DISCUSSION

### Prevalence of SHS at home, outside the home and inside the schools

This is the first study that describes SHS exposure at specific locations among a nationally representative sample of school-going adolescents in Malaysia. We found that more than one-third (37.8%) of the respondents were exposed to SHS at home. The prevalence of SHS exposure inside the home was higher than those reported in Saudi Arabia (32.7%)^18^ and among youth from 168 countries (30.4%)^19^. However, findings similar to ours were reported among youth in Gambia (38.2%)^12^. The latest prevalence of SHS exposure reported in our study was significantly lower compared with previous studies conducted in Malaysia. For instance, the Malaysian Global Youth Tobacco Survey (GYTS 2009) reported a prevalence of 48.7%^20^ while Lim et al.^14^ reported a prevalence of 56.4% among school-going adolescents in Malaysia. The reduction of SHS exposure was encouraging as the prevalence of smoking among Malaysian adults has plateaued since about a decade ago^21^. Higher level of awareness of smoking and SHS hazards among Malaysian adults might have caused them not smoke at home, leading to the reduction of SHS exposure at home among school-going adolescents. However, in-depth qualitative and quantitative studies are required to test our hypotheses. Enhancement of health promotion and smoke-free interventions among Malaysians are among the ways forward as both measures have shown promising efficacy in reducing SHS exposure inside the home^22^.

More than half (51.2%) of the respondents reported being exposed to SHS outside the home, which was similar to the prevalence reported by students in Saudi Arabia (49.3%)^18^. Another study conducted in 168 countries (44.2%)^19^ reported lower prevalence of SHS exposure outside the home. However, studies among youth from Gambia (61.4%)^12^ and Malaysia (2009; 64.1%) reported higher prevalence of SHS exposure compared to our findings^23^. Although the reduction of SHS exposure from 64.1% to 51.2% within 7 years is very encouraging, the prevalence of SHS exposure among Malaysian youth is still high. Therefore, more proactive measures such as expansion of smokefree public areas with comprehensive enforcement is needed. Adolescents should also be trained and encouraged to avoid SHS in public areas. The implementation of measures should involve all related stakeholders, parents, authorities, and community members, regardless of smoking status.

More than a quarter (27.3%) of school-going adolescents reported being exposed to SHS inside the school. The prevalence was higher than what had been reported by students in Gambia^12^ (21.3%) but lower than the prevalence reported in the GYTS conducted in the Federated States of Micronesia (65.7%), Mongolia (56.4%) and China (54.5%)^20^. The lower prevalence of SHS exposure in our study might be due to the introduction of smoke-free policies, which can create an anti-smoking norm inside the school. However, the prevalence of exposure in school was still high taking into consideration the prohibition of smoking in the school premises, which had been introduced two decades ago^9^. Our findings indicate the importance of cooperation among the stakeholders (school authority, health department and parent–teacher association) to tackle SHS exposure in the school. A study conducted in California reported lower risk of SHS exposure in school with stronger smoking regulations^24^. Therefore, health promotion and a full enforcement of s smoking ban should be carried out in a collaboration between the school and health authorities, as the way forward to reduce SHS exposure inside the school.

### Associated factors of SHS at home, outside the home and inside the schools

Our study found that male adolescents were at higher risk of SHS exposure outside the home and at school but less likely to be exposed to SHS in the home. Similar findings were reported among adolescents from Gambia, Saudi Arabia, and another 168 countries^12,18,19^, while a study among adolescents in Cambodia reported a non-significant difference between genders^17^. Studies revealed that boys are more active and spend more time in school and outdoor areas compared to girls^25^, which increases their risk for SHS exposure at these places. On the other hand, girls tend to be closer to their parents and stay at home more often, which increases their risk for SHS exposure at home rather than at outdoor areas^26^.

The odds of being exposed to SHS were higher among older adolescents at home, outside the home and in the school compared with younger adolescents. Similar findings were reported in Cambodia and Saudi Arabia^17,18^. A study among adults in Canada reported that those living with younger children tend to have smoke-free homes while those who were living with older children reported higher percentage of not having smoke-free rules in their homes^27^, which may be the plausible explanation for higher risk of SHS exposure among older adolescents at home in our study. Along with that, older adolescents spent less time with the family at home and have higher chance to be outside home, which increases their risk of SHS exposure at places they visit^28^.

Adolescents of Malay and Bumiputra Sarawak ethnicity were more likely to be exposed to SHS at home, outside the home and in the school compared with Chinese adolescents. Another study among Malaysian adolescents reported similar findings^14^. Our findings can be explained by the higher prevalence of smoking among adults of Malay, and Bumiputra Sarawak, ethnicity compared with Chinese, as adolescents living with smoking adults have a higher risk of being exposed to SHS^21^. In addition, a previous study among Malaysian adults reported that Malay adults were less likely to adopt a smoke-free home policy, which explains the higher likelihood of SHS exposure at home among Malay adolescents^29^.

Adolescents who were from rural areas were more likely to be exposed to SHS at home, outside the home and in the school. Other studies among adolescents from Gambia and Korea reported similar findings^12,26^. The Malaysian National Health and Morbidity Survey (2019) reported a significantly higher prevalence of smoking among adults residing in rural areas, which might have contributed to an increased risk of SHS exposure among adolescents from rural areas^21^. In addition, adolescents from rural areas had higher risk of SHS exposure possibly due to the higher prevalence of smoking among adolescents from rural areas^15^. It has been shown that having friends who smoke increases the risk of SHS exposure among adolescents^18^. Furthermore, the higher likelihood of SHS exposure in rural areas might be due to rural dwellers having less exposure to health promotion or anti-smoking campaigns^30^. This calls for more anti-tobacco programs and strict enforcement of tobacco control laws in the country, particularly in rural areas.

Our study showed that adolescents who were current smokers had higher odds of being exposed to SHS at home, outside the home and in the school. Our findings are consistent with those reported in studies among adolescents in Saudi Arabia, Gambia, and Malaysia^12,14,18^. These findings might have been expected in view of the fact that adolescents who smoke are less likely to perceive SHS as harmful and thus less likely to avoid SHS^31^. In addition, evidence suggested that smoking adolescents were more likely to have friends who smoked, compared to nonsmokers, and therefore have a higher risk of being exposed to SHS^32^.

It might have been expected that adolescents who had higher awareness of the harmful effects of SHS would have a lower risk of SHS exposure and vice versa. However, in our study, adolescents who did not perceive SHS as harmful had lower risk of being exposed to SHS outside the home and in the school, in contrast to what had been reported among adolescents in Saudi Arabia^18^. Nevertheless, our findings are in line with studies conducted among adolescents from South Africa, which reported that the perception of SHS as being harmful was associated with higher likelihood of SHS exposure^33^. Another recent study among adolescents from the United States similarly reported that those who thought more about the harmful effects of SHS had higher risk of SHS exposure, which is attributable to the fact that those who were exposed to SHS thought more about the harmful effects of SHS^34^. This phenomenon could also be due to the lack of SHS avoidance, as highlighted in a study in Jordan that avoidance behavior towards SHS can be low even with high awareness of the hazards of SHS^35^.

The Malaysian government is committed towards achieving the target of becoming a tobacco-free nation with all Malaysians being protected from SHS exposure by the year 2045 (endgame for tobacco)^5^. In achieving this target, several strategies have been implemented, including strengthening of existing tobacco control activities, strengthening of enforcement of national tobacco control legislation, empowerment of community, strengthening of multisectoral collaboration and strengthening of tobacco control activities based on the MPOWER strategy (Monitor tobacco use and prevention policies; Protect people from tobacco smoke; Offer help to quit tobacco use; Warn about the dangers of tobacco; Enforce bans on tobacco advertising, promotion and sponsorship; Raise taxes on tobacco). The implementation of such interrelated tobacco control measures has contributed to the reduction of SHS exposure at home and public places in Malaysia^5^.

### Strengths and limitations

This study is not without limitations. First, our study utilized cross sectional data and therefore causal relationships could not be established. Second, SHS exposure was based on self-reporting and thus there is the possibility of under-reporting or over-reporting due to recall bias. Despite these limitations, another study has proven that the self-report method can be reliable to measure SHS exposure^36^. In addition, our study is a nationwide study involving a representative sample, which enables generalization of the findings to adolescents in Malaysia.

## CONCLUSIONS

The findings of the present study provide information that is important, and needed, to inform policy making. Although many efforts have been undertaken to address the problem of SHS exposure among Malaysian adolescents, the prevalence of SHS exposure at home, outside the home and in the school is still high. Particular attention must be placed on planning programs and measures tailored to specific needs of adolescents identified to be at risk of SHS exposure. More health promotion activities and antismoking campaigns should be carried out to promote the adoption of smoke-free homes among Malaysian adults, to protect their children from SHS exposure at home. In addition to the expansion of smoke-free public places in Malaysia, strict and consistent enforcement of the smoking ban is needed to protect adolescent from being exposed to SHS at public places designated to be smoke-free zones. Also, smoking regulations in schools should be enhanced through collaboration between schools, parents, and health authorities.
